# A Separate-Pavilion Hospital

**Published:** 1923-08

**Authors:** 


### A SEPARATE-PAVILION HOSPITAL.

A NEW hospital has been opened at Jette-Saint-Pierre, near Brussels, by the King and Queen of the Belgians. It was built with the aid of a legacy left by the late Georges Brugmann to the Brussels Hospitals, with the object of founding a convalescent home for women, but, as a similar institution already existed at Verrewinkel-Uccle, the Hospital Council, with the consent of the Brugmann family, applied the legacy to building a new hospital with all modern conveniences. An agreement to this effect was drawn up in 1906, and the hospital was to have been finished in 1906, but various difficulties sprang up and the work was more or less suspended for a time. The unfinished buildings were used, during the War, for field hospitals and for War patients suffering from tuberculosis. At the end of the War, building was resumed, a great part being under the administration of the civil hospitals, and a few months saw the completion of the work of construction. M. Horta, Professor of Architecture at Brussels University, was chosen as architect, and before carrying out the work he visited the most important hospitals in France, England, Germany and Austria. His researches led him to adopt the principle of separate pavilions, mostly of only one storey, designed to obtain the maximum of isolation and fresh air, and surrounded by greenery. The whole building covers about 7 acres, and contains 650 beds. The Brugmann Hospital is to be the scientific centre of the University. At the opening ceremony M. Max, the famous Burgomaster of Brussels, said that the legacy of five million francs had been vastly exceeded by the expenses, and that the total cost of the hospital was 23,700,000 francs, of which 17,500,000 francs had already been paid off.

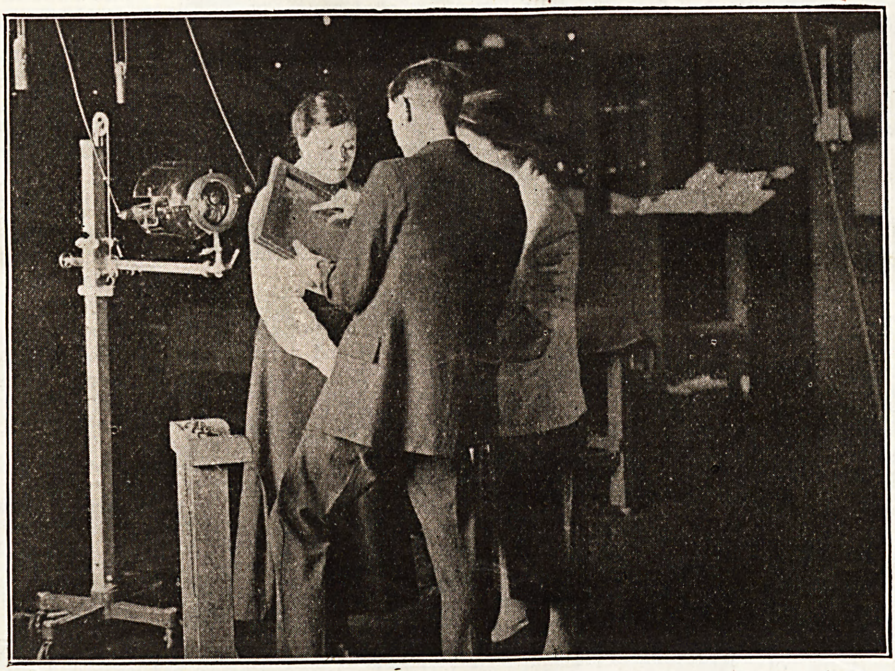

X-Ray Department Bridgwater Hospital: Screening.

### Alton Cottage Hospital.

Mr. B. W. Peile has resigned the post of hon. secretary and treasurer to the Alton Cottage Hospital which he has held for eighteen years. The committee has recommended that he be made a Life Governor.

